# Tempol Exerts Radioprotective Effects by Suppressing Radiation-Induced DNA Double-Strand Break Formation

**DOI:** 10.3390/ijms27062601

**Published:** 2026-03-12

**Authors:** Shinya Masugata, Megumi Sasatani, Tsutomu Shimura, Asako J. Nakamura

**Affiliations:** 1 Department of Biological Sciences, College of Science, Ibaraki University, Ibaraki 310-8512, Japan; 23nd105y@vc.ibaraki.ac.jp; 2 Department of Experimental Oncology, Research Institute for Radiation Biology and Medicine, Hiroshima University, Hiroshima 734-8553, Japan; mtoyosh@hiroshima-u.ac.jp; 3 Department of Environmental Health, National Institute of Public Health, Wako 351-0197, Japan; simura.t.aa@niph.go.jp

**Keywords:** γ-H2AX, ROS, inflammation, DNA double-strand break (DNA DSB), antioxidant

## Abstract

Concerns about radiation exposure following the Fukushima Nuclear Power Plant accident continue to grow, and health risks associated with medical radiation have also become an important issue. Therefore, identifying agents that can mitigate radiation-related health effects is necessary. We focused on the antioxidant 4-hydroxy-2,2,6,6-tetramethylpiperidine-N-oxyl (tempol) and investigated its radioprotective mechanisms. HeLa and TIG-3 cells were irradiated with X-rays, γ-rays, or heavy-ion beams. The effect of tempol on reactive oxygen species (ROS) production was evaluated using fluorescence-activated cell sorting (FACS) analysis. DNA double-strand break (DSB) formation was assessed by γ-H2AX immunofluorescence staining. In mice, γ-H2AX formation in the thymus and duodenum were evaluated after acute or chronic γ-ray exposure. Inflammatory responses were analyzed through macrophage infiltration and TNF mRNA expression, while apoptosis was measured using Annexin V staining. Tempol suppressed ROS production and γ-H2AX foci formation following irradiation. It also reduced γ-H2AX induction in mouse tissues. Activated macrophage infiltration and TNF expression in the duodenum tended to decrease in tempol-treated mice, whereas apoptotic levels showed no significant differences. Notably, tempol more effectively inhibited γ-H2AX formation during chronic irradiation than acute exposure. These findings suggest that tempol mitigates radiation-induced inflammation and reduces DNA damage, supporting its potential as a radioprotective agent.

## 1. Introduction

Ionizing radiation (IR) exposure has become increasingly common due to the widespread use of diagnostic imaging and radiation therapy in medicine [1], as well as the growing interest in space exploration. The Fukushima Daiichi Nuclear Power Plant accident has further heightened public concern about the health risks associated with radiation. Radiation can damage DNA either directly through ionization or indirectly via reactive oxygen species (ROS), leading to genomic instability, inflammation, and diseases such as cancer and cardiovascular disorders [2].

Among the various types of DNA damage, double-strand breaks (DSBs) are particularly severe, as they compromise genomic integrity and are difficult to repair [3,4]. Suppressing DSB formation is therefore critical for radiation protection. Since ROS-mediated DSBs can be reduced by scavenging ROS, antioxidants have emerged as promising protective agents [5]. Amifostine, an FDA-approved antioxidant, has shown protective effects but is limited in clinical use due to adverse side effects [6].

Radiation exposure varies in type and context. X-rays and γ-rays primarily cause DNA damage through ROS, while heavy particle beams and α-rays induce more complex and direct DNA damage that is harder to repair [7]. Additionally, the biological effects of radiation differ depending on dose rate; acute high-dose exposure has different consequences compared to chronic low-dose exposure [8,9]. Thus, protective agents should be effective across various radiation types and exposure scenarios.

4-hydroxy-2,2,6,6-tetramethylpiperidine-N-oxyl (tempol) is a synthetic antioxidant that mimics superoxide dismutase (SOD) and has shown potential as a radioprotective agent [10]. Studies have shown that tempol reduces mortality and cancer incidence in irradiated mice, and suppresses micronucleus formation and chromosomal aberrations [11,12]. However, few studies have directly compared the effects of tempol on DNA damage induced under different radiation types and irradiation conditions. Moreover, the mechanisms underlying its protective effects remain unclear.

Radiation-induced DNA damage activates biological responses such as inflammation, apoptosis, and cellular senescence. Inflammation is mediated by NF-κB activation through p53 and ATM signaling, leading to cytokine production [13]. ROS further amplifies inflammation via NF-κB and MAPK pathways, contributing to chronic tissue damage and carcinogenesis [14]. Although these findings shows that tempol exsert the suppressive effect against radiation-induced inflammation, the effect of tempol remain unclear.

Apoptosis, while essential for cellular homeostasis, also contributes to acute tissue injury. In the process, phosphatidylserine in the cell membrane is exposed to the outside of the cell and sends phagocytosis signals to macrophages [15]. And then, the apoptotic cells are eventually phagocytosed by activated macrophages. During macrophage activation, monocytes express the membrane protein F4/80 and infiltrate tissues by acquiring macrophage functions [16]. Therefore, apoptosis can be linked to inflammatory responses.

This study aims to clarify tempol’s radioprotective mechanisms by examining its effects on radiation-induced DSB formation, inflammation, and apoptosis. The findings will enhance understanding of tempol’s role in mitigating radiation-induced biological damage and support its potential as a therapeutic agent.

## 2. Results

### 2.1. Tempol Suppresses the ROS Production and Irradiation-Induced DNA Damage In Vitro

**Figure 1:**
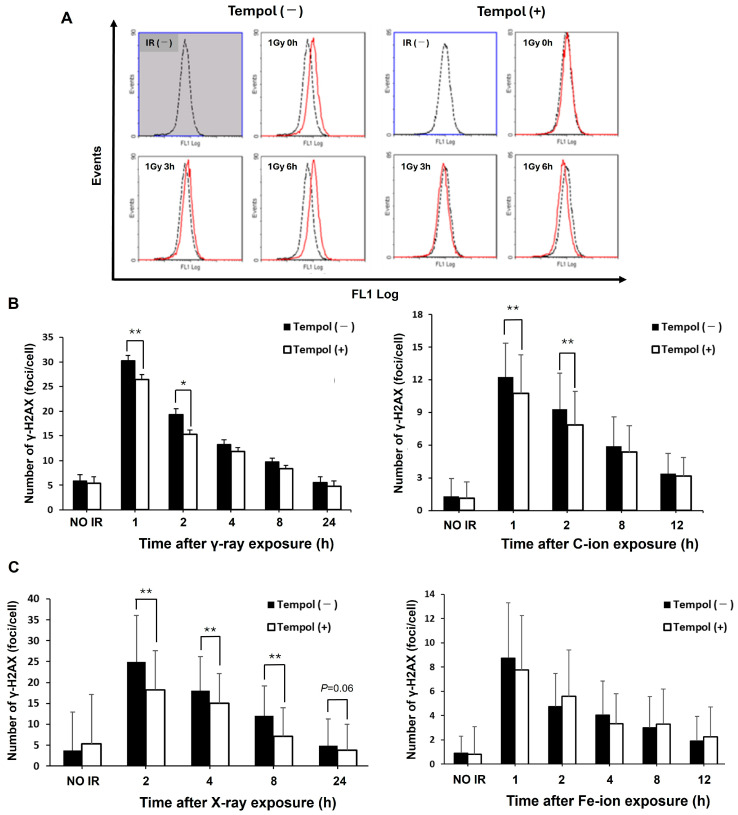
Radiation-induced ROS production and DSB formation are suppressed in tempol-treated cells. HeLa or TIG-3 cells were cultured in medium containing 50 µmol/L tempol for 1 week and irradiated with 1 Gy γ-rays or particle beams. (**A**) Intracellular ROS levels in HeLa cells until 6 h after 1 Gy radiation. (**B**) Average number of γ-H2AX foci per cell in HeLa cells after 1 Gy of γ-ray or C-ion exposure. Error bars indicate standard deviation. (γ-ray: *n* ≥ 100, C-ion: *n* ≥ 50) * *p* < 0.05 ** *p* < 0.01. (**C**) Average number of γ-H2AX foci in TIG-3 cells after X-ray and Fe-ion irradiation. Error bars indicate standard deviation. (X-ray: *n* ≥ 100 Fe-ion: *n* ≥ 50) ** *p* < 0.01.

We first evaluated the effects of tempol on ROS production and subsequent DNA damage induced by radiation in cultured cells. The number of HeLa cells with high ROS levels decreased in the tempol-treated group compared to untreated cells immediately after 1 Gy of γ-ray irradiation until 6 h (Figure 1A). This result suggested that tempol can suppress ROS production even under low-concentration treatment conditions. The number of γ-H2AX (DSB marker) foci per cell was significantly reduced in the tempol-treated cells up to 2 h after 1 Gy irradiation (γ-ray 1 h vs. γ-ray 1 h + tempol, *t* = 2.7, *p* < 0.01) (Figure 1B). Next, we evaluated the protective effect of tempol against particle radiation, anticipated in future space exploration. Tempol suppressed γ-H2AX levels in carbon ion-irradiated cells for several hours after irradiation (C-ion 1 h vs. C-ion 1 h + tempol, *t* = 3.3, *p* < 0.01) (Figure 1B). Furthermore, in TIG-3 cells, Tempol reduced γ-H2AX formation induced by X-ray 1 Gy (X-rays 2 h vs. X-rays 2 h + tempol, *t* = 6.5, *p* < 0.01) (Figure 1C). Its findings demonstrate that tempol significantly reduces the formation of γ-H2AX foci following radiation exposure in TIG-3 cells, indicating its radioprotective effect in normal cells. Moreover, although the significant difference is not observed, γ-H2AX induced by iron ion is slightly reduced in tempol-treated TIG-3 cells against control cells at 1 h after irradiation (Fe-ion 1 h vs. Fe-ion 1 h + tempol: 8.8 vs. 7.7, *p* > 0.05) (Figure 1C). These results suggest that tempol primarily suppresses DNA damage induced via ROS generation.

### 2.2. Inhibitory Effect of Tempol on Radiation-Induced DNA Damage in Biological Tissues

**Figure 2:**
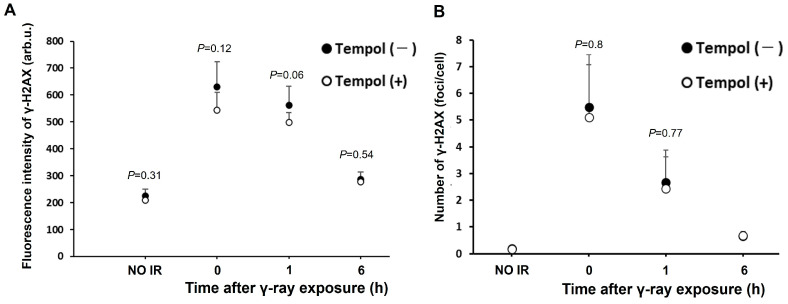
Tempol suppresses radiation-induced DSB formation in mouse tissues. Mice were fed either a normal diet or a diet containing Tempol (10 mg/g) for 5 d, followed by exposure to 1 Gy γ-rays. The thymus and duodenum of the mice were collected at the following time points: immediately after irradiation (0 h), 1 h and 6 h. (**A**) Fluorescence intensity of γ-H2AX in mouse thymus after 1 Gy γ-ray irradiation. Error bars indicate mean ± SEM from two independent experiments using a total of 3–4 mice (1Gy-1hr: *n* = 4, others: *n* = 3). arb.u.: arbitrary unit. (**B**) Average number of γ-H2AX foci per cell in mouse duodenum after 1 Gy γ-ray irradiation. Error bars indicate standard deviation. (1Gy-1hr: *n* = 4, others: *n* = 3). *p*-value indicates significant differences compared with Tempol (−).

Next, we evaluated the effects of tempol on radiation-induced DNA damage in mouse tissues. In this study, we focused on the immune and digestive systems specifically the thymus and duodenum, which are vulnerable to radiation-induced injury. To assess DNA damage in the thymus cells, we performed FACS analysis to measure γ-H2AX levels. Administration of tempol tended to suppress the increase in γ-H2AX fluorescence intensity in mice for up to 6 h following 1 Gy irradiation. (γ-rays 1 h vs. γ-rays 1 h + tempol: 562 vs. 499, *t* = 2.1, *p* = 0.06) (Figure 2A). Immunostaining for γ-H2AX levels in duodenum tissue sections revealed a reduction in the number of γ-H2AX foci per cell in tempol-treated mice compared to the control group, observed up to 1 h post-irradiation (γ-rays 0 h vs. γ-rays 0 h + tempol: 5.5 foci/cell vs. 5.1 foci/cell, *p* > 0.05) (Figure 2B). Thus, tempol may inhibit radiation-induced DNA damage in vivo.

### 2.3. Inhibitory Effect of Tempol on Radiation-Induced Inflammation in Biological Tissues

**Figure 3:**
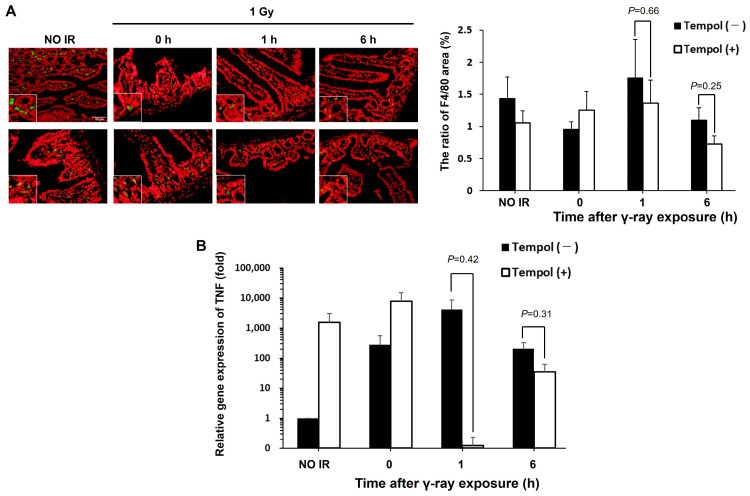
Tempol suppresses the inflammatory response in mouse tissues after radiation exposure. (**A**) Macrophage activation in mouse duodenum after 1 Gy whole-body γ-ray irradiation. (**Left**) Representative image of F4/80 staining. Scale bar: 50 µm (**Right**) Graph of F4/80 fluorescence area. The error bar indicates standard error. (*n* = 3) (**B**) Gene expression of TNF in the mouse duodenum after 1 Gy γ-ray irradiation.

Tissue inflammation contributes to the progression from radiation-induced DNA damage to final disease development. Thus, we evaluated the effect of tempol on radiation-induced inflammation in the mouse duodenum. We found that tempol has an inhibitory effect on the formation of DNA damage after γ-ray irradiation on Figure 2. First, we focused on macrophages, activated during inflammation, and evaluated the number of activated macrophages following radiation exposure by immunofluorescence staining. The number of F4/80-positive cells, a marker of activated macrophages, decreased in mice duodenum treated with tempol after 1 h of radiation exposure compared to that in non-treated mice, although the significant difference was not observed. (γ-rays 1 h vs. γ-rays 1 h + tempol: 1.75% vs. 1.36%, *t* = 0.50, *p* > 0.05) (Figure 3A). Next, we examined the expression of tumor necrosis factor (TNF), an inflammatory cytokine secreted by macrophages that promote inflammation, using RT-qPCR. An increase in TNF gene expression, similar to macrophage activation, 1 h after irradiation tended to be suppressed in mice treated with tempol compared to non-treated mice (γ-rays vs. γ-rays + tempol: 4143 vs. 0.12, *t* = 1.00, *p* > 0.05) (Figure 3B). These results suggest that tempol may inhibit radiation-induced inflammation in biological tissues.

### 2.4. Effect of Tempol on Radiation-Induced Apoptosis

Tempol may inhibit radiation-induced inflammation. When apoptotic cells are phagocytosed by activated macrophages, the inflammatory response switches to an anti-inflammatory response [17]; the inflammatory response is sustained with several apoptotic cells. Then, the anti-inflammatory effects of tempol on radiation-induced inflammation may indicate that tempol inhibits radiation-induced apoptosis.

**Figure 4:**
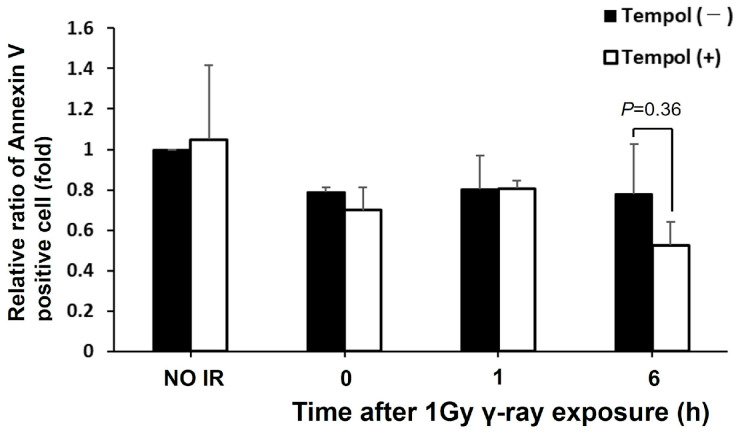
Tempol does not inhibit radiation-induced apoptosis. The percentage of Annexin V-positive cells in the mouse duodenum until 6 h after 1 Gy γ-ray irradiation. The vertical axis of the graph shows the relative value compared to 0 Gy (NO IR) and tempol (−). The error bar shows the standard error. (*n* = 3).

The effect of tempol on radiation-induced apoptosis was evaluated in the mouse duodenum. Annexin V, which binds to phosphatidylserine, was used as an apoptotic marker for immunostaining. No increase in Annexin V levels after radiation exposure and no difference between the control and tempol-administered groups were observed (γ-rays 6 h vs. γ-rays 6 h + tempol: 0.67 vs. 0.44, *p* > 0.05) (Figure 4). Furthermore, the weight of thymus showed no significant difference between the control and tempol-treated groups. These results suggest that apoptosis was not induced by the dose or at the time points studied.

### 2.5. The Effect of Tempol on DNA Damage Induced by Chronic Exposure to Radiation

**Figure 5:**
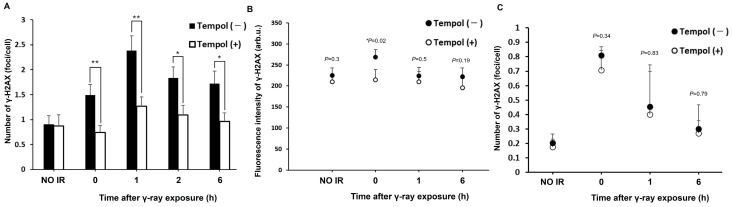
Tempol suppresses DSB formation after chronic irradiation. (**A**) HeLa cells were cultured in medium containing 50 µmol/L tempol for 1 week, followed by fixing and staining for γ-H2AX at 0, 1, 2, and 6 h after 1 Gy γ-ray irradiation. The graph shows the mean number of γ-H2AX foci. Error bars indicate standard deviation (*n* ≥ 100) * *p* < 0.05, ** *p* < 0.01. (**B**,**C**) Mice were fed either a normal diet or a diet containing tempol and exposed to 1 Gy γ-rays (0.694 mGy/min) for 24 h. Tissue samples were obtained from mice at 0 h, 1 h, and 6 h. The graph shows the fluorescence intensity of γ-H2AX in the mouse thymus B. and the average number of γ-H2AX foci in the mouse duodenum after chronic γ-ray irradiation C. The error bars indicate mean ± SEM from two independent experiments (**B**) or the standard deviation (**C**). (1Gy-1hr: *n* = 4, others: *n* = 3) *p-*value indicates significant differences compared with Tempol (−). * *p* < 0.05. arb.u.: arbitrary unit.

Tempol may have a protective effect against the harmful effects of acute radiation exposure. So, we evaluated the protective effect of tempol under chronic irradiation conditions (0.694 mGy/min) because chronic exposure in nuclear accident or in space is a matter of concern. Tempol significantly suppressed DNA damage compared to untreated cells over a 6 h period after radiation exposure (γ-rays 6 h vs. γ-rays 6 h + tempol, *t* = 2.5, *p* < 0.05) (Figure 5A). A similar decreasing trend was observed in the mouse tissues (thymus: γ-rays 0 h vs. γ-rays 0 h + tempol, *t* = 2.8, *p* < 0.05) (Figure 5B,C). Its suppressive effect was more remarkable than that of acute radiation exposure. These results suggest that tempol may suppress the induction of DNA damage by radiation under chronic exposure conditions.

## 3. Discussion

In this study, we first showed that tempol have inhibitory effects against radiation-induced DNA damage following acute radiation exposure under in vitro and in vivo conditions. Next, we showed the suppressive effect of tempol against the inflammatory response including the macrophage activation and the gene expression of inflammatory cytokines in the duodenum of mice. In contrast, tempol did not suppress radiation-induced apoptosis. Finally, we elucidated that tempol reduced DNA damage under particle beam and chronic γ-ray irradiation in vitro.

Among the various types of radiation, DNA damage induced by X-rays and γ-rays used in medical treatments is mainly indirect, due to ROS production, an important target for protection against radiation. Tempol, a powerful antioxidant, rapidly scavenges ROS and detoxifies [18]. Then, tempol is expected to be useful as a radioprotective agent, and several previous studies have reported its protective effects against radiation [12,19]. In this study, we first evaluated the effect of tempol on ROS levels and DNA damage after radiation exposure and found that it suppressed ROS production and DSB formation both in vitro and in vivo systems, even several hours after γ-ray irradiation. Tempol significantly inhibited DNA damage in chronically irradiated cells. Furthermore, we evaluated the effect of tempol on DNA damage induced by high-LET radiation using carbon- and iron-ion. These radiations induced γ-H2AX foci less than γ-ray. These results support the report that high-LET radiation induces a few clustered DNA damages compared to low-LET radiation [20]. As a result, tempol showed a similar inhibitory effect on carbon-ions. In contrast, iron-ion irradiation, which has a more direct effect than carbon-ion, the inhibitory effect of tempol is not observed. These results suggested that the inhibitory effect of tempol on radiation-induced DNA damage can be attributed to ROS removal via its antioxidant action. It has been reported that high-LET radiation such as iron ion induces DNA damage via ROS generation as well as low-LET radiation [21]. Furthermore, approximately 60% of DNA damage induced by high-LET radiation is attributed to ROS generation [22]. In fact, tempol showed the suppressive effect on carbon-ion induced DNA damage in this study (Figure 1B). However, the type of ROS induced by high-LET radiation differs from that induced by low-LET radiation. High-LET radiation primarily generates hydroxyl radicals, which are highly reactive with DNA. Tempol primarily suppresses superoxide, the predominant ROS produced by low-LET radiation [23,24]. Accordingly, we think that the significant suppression of γ-H2AX foci induced by Fe-ion is not observed, since the effect of scavenging of ROS by tempol is small.

ROS have been implicated in DNA damage, and recruitment of monocytes and their differentiation into macrophages, thereby inducing a tissue inflammatory response [25]. In the tissue inflammatory response, macrophages infiltrate and are activated within the tissue, thereby contributing to the induction of inflammation. Tempol suppresses the activation of NF-κB and reduces the gene expression of TNF-α in mice with carrageenan-induced pleurisy [26]. Consequently, if tempol exerts an inhibitory effect on radiation-induced inflammation, this may contribute to the mechanism of the radioprotective effect of tempol demonstrated in previous studies, as chronic radiation-induced inflammation can change the tissue microenvironment and directly contribute to disease development, including cancer.

In this study, we evaluated the effect of tempol on macrophage activation by performing immunofluorescence staining of F4/80-positive macrophages in the mouse duodenum at several time points (0 h, 1 h, 6 h) after radiation exposure. In previous research, apoptosis is markedly induced 4–6 h after exposure to 1 Gy ionizing radiation in mouse small intestine [27]. The results suggested suppression of infiltration and activation of macrophages in the tempol-treated group compared to those in the non-treated group at 1 h and 6 h after radiation exposure. These results support the observation that tempol suppresses neutrophil migration and infiltration under inflammatory conditions [28,29,30,31]. Thus, tempol may suppress radiation-induced inflammatory responses mediated by macrophage activation.

In contrast, in samples collected immediately after irradiation, the number of activated macrophages increased in tempol-treated mice, suggesting that tempol may accelerate the onset of inflammation. Tempol may activate a rapid inflammatory response after irradiation while suppressing the chronicity of inflammation. Chronic inflammation promotes tumor formation in tissue [32,33]. During chronic inflammation, ROS released by immune cells, such as macrophages, damage the DNA of surrounding cells and induce genomic instability [34,35]. Then, if Tempol suppresses activation of macrophages after 1 h of radiation exposure and the subsequent ROS production, it may contribute to the suppression of radiation-induced inflammation. To assess whether tempol affects the gene expression of inflammatory cytokines during inflammatory reactions, we evaluated the gene expression of TNF, which is released from macrophages and promotes inflammatory reactions. In the non-treated group, a strong increase in the gene expression was observed 1 h after radiation exposure. In contrast, in the tempol-treated group, the TNF expression was highest immediately and 1 h after radiation exposure; then, TNF expression was strongly suppressed. This result is consistent with those of activated macrophage expression, and tempol may accelerate the induction and resolution of radiation-induced inflammation at the gene level.

During radiation-induced inflammation, the acute inflammation sustained 24 h after irradiation, so the irradiated samples used in this study may correspond to the acute inflammation phase [36]. The results in this study suggest that radiation-induced DNA damage was suppressed in the tempol-treated group compared to the non-treated group, resulting in reduced cell death or apoptosis, or a rapid induction of acute inflammation, which subsided, thereby suppressing persistent inflammation.

Cell death, including apoptosis, is a biological response closely related to the inflammatory response in the tissue microenvironment, which can be due to radiation. We evaluated the effect of tempol on radiation-induced apoptosis following radiation-induced inflammation. Specifically, we performed immunostaining for Annexin V as a marker in the duodenum specimens used to assess radiation-induced inflammation. There was no increase in the level of Annexin V positive cells after radiation exposure, and the non-treated and tempol-treated groups did not differ significantly. Although these results suggest that tempol may inhibit radiation-induced apoptosis, evaluating its effect on apoptosis in the early stages after radiation exposure may not be possible.

Although no significant difference was observed, these data showed a slight reduction in the Annexin V levels in the tempol-treated group compared to the non-treated group at 6 h after radiation exposure.

Tempol activates the PI3K/Akt/Nrf2 pathway 24 h after acetaminophen administration in mice with acetaminophen-induced acute liver toxicity when administered in advance, showing anti-apoptotic effects [37]. Furthermore, tempol exerts a similar effect in the kidney 24 h after renal ischemia/reperfusion injury via the activation of the PI3K/Akt/Nrf2 pathway [38]. Moreover, in radiation-induced apoptosis, Tempol reduced apoptosis in TK6 cells 48 h following 6Gy radiation exposure through activating MAPK/ERK pathway [39]. Accordingly, evaluating the anti-apoptotic effect of tempol by analyzing the tissue at 6 h or later after radiation exposure may be possible.

We showed that tempol suppressed the infiltration of F4/80-positive macrophages and the gene expression of TNF induced by radiation. This supports previous findings showing that tempol suppresses the infiltration of F4/80-positive macrophages in mice with renal fibrosis and the expression of TNF-α in hepatocytes under inflammatory conditions and expression of TNF genes underlying microglial activation [40,41,42].

Another reason for the anti-inflammatory effect observed in the mouse duodenum after radiation exposure is the membrane permeability of tempol, a small molecule that easily penetrates cells. In this study, tempol powder was mixed with solid feed and administered to facilitate its direct absorption by duodenal epithelial cells during the digestion of tempol-containing feed in the duodenum. An antioxidative environment is constantly maintained within the tissue; thus, the radioprotective effects are more likely. In contrast, a tendency of DSB suppression was observed in the thymus tissue, distant from the digestive organs, after radiation exposure. Thus, if tempol is administered continuously for several days, it is likely that it is taken up by remote organs via the bloodstream, exerting a protective effect.

The anti-inflammatory effect of tempol is among the ROS removal mechanisms [17,23]. Tempol may exert a protective effect on cells and tissues by directly removing ROS and modulating intracellular signals [42,43]. Nuclear factor E2-related factor 2 (Nrf2), a transcription factor, is central in antioxidant responses. When activated, Nrf2 migrates into the nucleus and induces the expression of antioxidant enzymes, such as heme oxygenase-1 (HO-1) [44]. Nrf2 suppresses ROS-induced NLRP3 inflammasome formation and reduces the gene expression of the inflammatory cytokine IL-1β [45]. It is among the mechanisms underlying tempol’s anti-inflammatory action. Tempol promotes cell survival by activating the PI3K/Akt pathway and inhibits apoptosis in glomerular epithelial cell lines by inhibiting the poly (ADP-ribose) polymerase-1 (PARP-1) signaling pathway [38,46]. Then, tempol may exert anti-inflammatory and anti-apoptotic effects along with other intracellular signal transduction factors. The identification of the signal transduction factors with which tempol interacts and the elucidation of their mechanisms of action are necessary to comprehensively understand the mechanism of tempol’s radioprotective effects.

The biological effects of acute (400 mGy/min) and chronic (15 mGy/min) radiation exposure is different [47]. In this study, tempol showed a significant inhibitory effect on DNA damage in chronically irradiated cells until 6 h after radiation exposure, both in vitro and in vivo. In particular, in the in vitro experiments, DNA damage was significantly suppressed at all time points up to 6 h after radiation exposure. This suggests that the contribution of ROS to DNA damage under chronic irradiation may be greater than that under acute irradiation. These results suggest that tempol may exert stronger radioprotective effects during chronic exposure.

Moreover, normal cells and cancer cells were used in this study. Tempol showed the suppressive effects for radiation-induced ROS generation and DSB formation in cancer cells. Tempol has been applied to radiotherapy patients in clinical settings as radioprotective agents [48,49]. At that time, the radioprotective effect for cancer cells should be minimized. On this point, cancer cells are exposed to hypoxic microenvironments in the body, several studies have indicated that the radioprotective effects of tempol may be limited under such conditions [23,50]. In addition, it has demonstrated that tempol can increase ROS levels specifically in cancer cells and subsequently inhibit their proliferation [51,52]. These studies suggest that tempol has been proven to be highly safe as a radioprotective agent.

Furthermore, tempol treatment at the low concentration used in this study (50 µM) suppressed DNA damage in HeLa cells; however, compared with TIG-3 cells, the duration of significant suppression was shorter (HeLa: 2 h vs. TIG-3: 4 h), and the level of suppression was smaller. These results support the findings that tempol exerts its protective effects preferentially in normal cells rather than in cancer cells.

In this study, the number of mice is only three in each experimental group, since practical limitations are present due to the large number of time points and conditions we aimed to analyze. Consequently, statistical reliability is limited in this study. In this context, further validation of the effects of tempol on radiation-induced biological responses is warranted in future investigations. Nevertheless, we believe that the data presented here provides meaningful insights and serves as a valuable foundation for future research.

In summary, we showed that tempol scavenged ROS produced by radiation and reduced DNA damage following acute and chronic irradiation. Furthermore, tempol may exert a protective effect against high-LET radiation such as carbon ions, as well as X-rays and γ-rays. Tempol protects against radiation under various exposure situations, including different types and dose rates of radiation, such as in radiation therapy and high-LET radiation in space. Tempol can suppress biological responses, such as radiation-induced tissue inflammation. Thus, tempol may exert a radioprotective effect on early biological responses after radiation exposure at the tissue level, suggesting that it may exert a protective effect against acute radiation damage in addition to radiation-induced diseases. Clarification of tempol’s radioprotective action can facilitate its use more safely and effectively as a radioprotective agent in modern society medicine in the future.

## 4. Materials and Methods

### 4.1. Cell Culture and Radiation Exposure

HeLa cells were obtained from the ATCC (Manassas, VA, USA). The cell line was cultured in DMEM supplemented with 10% FBS and maintained in a humidified incubator at 37 °C with 5% CO_2_ and 20% O_2_. One week before radiation exposure, the culture medium was replaced with a medium containing 50 µM tempol (Wako Pure Chemical Industries, Ltd., Osaka, Japan). The culture with the medium containing tempol was maintained until irradiation. One day before irradiation with 1 Gy of ^137^Cs γ-rays (1 Gy/min) using a Gammacell 40 Exactor (MDS Nordion, Ottawa, ON, Canada), the cells were trypsinized and seeded onto LabTeck 2-well chamber slides (Nunc, Waltham, MA, USA). The irradiation experiment was performed in a circle with a diameter of 10 cm in chamber. The source-skin distance (SSD) was assumed to be about 38 cm by the statement in previous study [53].

For chronic irradiation, cells were irradiated with 1 Gy using a low-dose-rate irradiation device (Industrial Scientific, Tokyo, Japan) at the Research Centre for Radiation Biology and Medicine, Kyoto University (source: ^137^Cs γ-rays, dose rate: 0.652 mGy/min). During low dose irradiation, cells were maintained in a humidified incubator at 37 °C with 5% CO_2_, and 20% O_2_. For heavy-dose irradiation, cells were irradiated with carbon ions (LET: 14 keV/µm) at 1 Gy and iron ions (LET: 200 keV/µm) at 1 Gy using the Heavy Ion Medical Accelerator in Chiba (HIMAC, Toshiba, Kanagawa, Japan) at the National Institute of Radiological Sciences. The irradiation experiment was performed in a field with a diameter of 10 cm. The cells were irradiated at the center of spread-out Bragg peak (SOBP), which is located 3 cm upstream from the maximum range of beams.

Human fetal lung fibroblast TIG-3 cells were purchased from the JCRB Cell Bank (Osaka, Japan) of the National Institute of Biomedical Innovation. TIG-3 cells were cultured under the same conditions as HeLa cells and treated with tempol for one week. The cells were seeded in a 35 mm dish 1 day before irradiation. On the next day, the cells were irradiated with 1 Gy of X-rays (2.0 Gy/min) using an X-ray irradiation device (OM-B205; Ohmic Co., Tokyo, Japan).

### 4.2. Animal Model and Irradiation Conditions

Seven-week-old male C57BL/6NCr(B6) mice were obtained from Charles River Japan, Inc., Kanagawa, Japan. The mice were kept in autoclaved cages lined with sterilized wood chips in a room with a controlled temperature (24 ± 2) °C, humidity (55 ± 10)%, and a 12 h light/12 h dark cycle, following the ‘Guidelines for the Care and Use of Laboratory Animals’ established by Hiroshima University. The mice were allowed to free access commercial MF feed (Oriental Yeast Co., Tokyo, Japan) and tap water until irradiation. All experiments were performed on animals from the same lot. Five days before irradiation, mice in the tempol-treated group were switched to a diet containing 10 mg tempol per gram of feed. The intake of tempol per day was estimated to be about 800–1000 mg/kg per mouse. The mice were sacrificed at multiple time points, and tissues were collected. We focused on thymus and duodenum, that are sensitive to radiation exposure.

### 4.3. Radiation Exposure in Mice

Each group of mice (3 mice per group) was placed in a box and exposed to radiation. Acute radiation exposure was conducted using a Gammacell 40 Exactor (source: ^137^Cs γ-rays, 148 TBq) (Best Theratoronics, Ottawa, ON, Canada), and chronic irradiation device (source: ^137^Cs γ-rays, 1.11 TBq) (Sangyo Kagaku, Tokyo, Japan) exposure in the irradiation area of the Research Institute for Radiation Biology and Medicine at Hiroshima University. The mice were held in a columnar container with a diameter of 10 cm. SSD was estimated to be about 35 cm [53]. The dose rate in acute irradiation was 1 Gy/min, and that in chronic irradiation was 0.694 mGy/min. The mice were sacrificed by either inhalation and tissues were collected for analysis. The body and tissue weights of all mice were measured.

### 4.4. Immunostaining

Immunostaining was performed using tissue sections to evaluate the levels of γ-H2AX, F4/80 and Annexin V. HeLa and TIG-3 cells were fixed in 2% paraformaldehyde (PFA) (γ-H2AX staining) or 4% PFA (F4/80, Annexin V staining) at room temperature for 20 min, and slides were immunostained with mouse monoclonal anti-γ-H2AX antibody (1:500; 05-636, MERCK Millipore, Burlington, MA, USA). Mouse duodenum was frozen in liquid nitrogen immediately after collection and stored at −80 °C until slide preparation. The frozen tissue was embedded in a Tissue-Tek boat filled with the Optimal Cutting Temperature (OCT) compound and frozen in liquid nitrogen-cooled isopentane. The tissue block was cut into sections of 4 µm thickness using a CM1860 cryostat (Leica Biosystems, Nuss Loch, Germany), attached to a glass slide, and air-dried at room temperature. Tissue slides were immersed in PBS at room temperature for 15 min to perform hydrophilic treatment. They were then fixed with 2% paraformaldehyde for 20 min and immunostained with rabbit polyclonal anti-γ-H2AX antibody (1:500; NB100-368, Novus, Centennial, CO, USA), rat monoclonal anti-F4/80 antibody (1:500; ab6640, abcam, Cambridge, UK) and rabbit polyclonal anti-Annexin V antibody (1:200; ab14196, abcam, Cambridge, UK), respectively. Following wash with PBS, the cells were stained Alexa Flour 488 Goat anti-rabbit IgG (1:700; A11034, Invitrogen, Carlsbad, CA, USA) and Alexa Flour 488 Goat anti-rat IgG (1:700; A11006, Invitrogen, Carlsbad, CA, USA), respectively. Subsequently, double-stranded RNA was degraded using RNase A solution (500 µg/mL; 740505, MACHEREY-NAGEL, Duren, North Rhine-Westphalia, Germany), and the cell nuclei were counterstained with propidium iodide. The stained slides were observed and photographed using an Olympus BX40 and BX53 microscope with Olympus DP73 camera (Olympus, Tokyo, Japan). The γ-H2AX foci were visually counted.

### 4.5. Fluorescence-Activated Cell Sorting (FACS) Analysis

The thymus was fixed by 70% Et-OH after collected from mice. After thymus was dissolved, the cells were washed with PBS containing 1% FBS and 0.01% NaN3 1 mL twice. After that, the cells were centrifuged 5000 rpm for 3 min. Next, the cells were permeabilized with 0.5% triton-X and immunostained with anti-γ-H2AX antibody 50 µL (1:100). Following the standing on ice for 20 min, the cells washed with 0.1% triton and immunostained with Alexa Fluor 488 Goat anti-rabbit IgG 40 µL (1:50). After two PBS washes, the cell nuclei were counterstained with propidium iodide (1:2). The fluorescence intensity of γ-H2AX was measured for each cell using a flow cytometer (FACS Canto II BD) (BD Biosciences, Franklin Lakes, NJ, USA) at excitation and emission wavelength of 488 nm and 530 nm, respectively.

### 4.6. ROS Detection

HeLa cells were treated with 20 µmol/L 2,7-dichlorofluorescin diacetate (DCFH-DA) for 30 min, and the intracellular ROS level was measured by FACS analysis to evaluate the effect of tempol on radiation-induced ROS production. Analyses were performed using at least 100 cells for each condition.

### 4.7. Evaluation of F4/80-Positive Regions

Evaluation of F4/80-positive regions was performed by immunostaining using tissue sections. Tissue slides were immersed in PBS for 15 min and fixed with 2% paraformaldehyde for 20 min. Next, they were immunostained with rat monoclonal anti-F4/80 antibody (1:500; ab6640, abcam, Cambridge, UK) and Alexa Flour 488 Goat anti-rat IgG (1:700; A11006, Invitrogen, Carlsbad, CA, USA), respectively.

The analysis was performed based on digital images acquired using an Olympus BX53 fluorescence microscope. The digital image of F4/80 was acquired using an objective lens of ×40. The ratio of the F4/80-positive area to the propidium iodide-stained area (cell nucleus) per field of view (FOV) for each duodenum sample was calculated using ImageJ (Fiji-win32 downloaded from https://fiji.sc/, accessed on 12 March 2026).

### 4.8. qRT-PCR

Total RNA was isolated from frozen OCT-composite sections using the RNeasy Plus Micro Kit (Qiagen, Hilden, Germany) following the manufacturer’s protocol. Reverse transcription was performed using SuperScript IV VILO Master Mix following the manufacturer’s protocol with ezDNase (Invitrogen, Carlsbad, CA, USA). Quantitative polymerase chain reaction (qRT-PCR) was analyzed in triplicate using gene expression assays (Thermo Fishier Scientific, Waltham, MA, USA). Relative expression was quantified using the 2^−ΔΔCt^ method. TNF gene is targeted in this analysis. The expression level of the TNF gene was normalized by GAPDH mRNA expression. The following primer sets were used: TNF, Mm00443258_m1; GAPDH, Mm99999915_g1.

### 4.9. Statistical Analysis

The data for γ-H2AX immunofluorescence staining (γ-irradiation) are expressed as mean standard deviation (SDM). The data for F4/80 and Annexin V immunofluorescence staining and RT-qPCR are expressed as mean ± standard error of the mean (SEM). The significance of the data was evaluated using the *F-* and *t-*tests. We performed Student’s *t-*test (two-tailed) or Welch’s *t-*test (two-tailed) based on the results of the *F* test. We considered the results as statistically significant for *p-*value < 0.05.

## Data Availability

The data will be shared on reasonable request to the corresponding author.
